# Requirements for Efficient Proteolytic Cleavage of Prelamin A by ZMPSTE24

**DOI:** 10.1371/journal.pone.0032120

**Published:** 2012-02-15

**Authors:** Jemima Barrowman, Corinne Hamblet, Megan S. Kane, Susan Michaelis

**Affiliations:** Department of Cell Biology, The Johns Hopkins School of Medicine, Baltimore, Maryland, United States of America; Omaha Veterans Affairs Medical Center, United States of America

## Abstract

**Background:**

The proteolytic maturation of the nuclear protein lamin A by the zinc metalloprotease ZMPSTE24 is critical for human health. The lamin A precursor, prelamin A, undergoes a multi-step maturation process that includes CAAX processing (farnesylation, proteolysis and carboxylmethylation of the C-terminal CAAX motif), followed by ZMPSTE24-mediated cleavage of the last 15 amino acids, including the modified C-terminus. Failure to cleave the prelamin A “tail”, due to mutations in either prelamin A or ZMPSTE24, results in a permanently prenylated form of prelamin A that underlies the premature aging disease Hutchinson-Gilford Progeria Syndrome (HGPS) and related progeroid disorders.

**Methodology/Principal Findings:**

Here we have investigated the features of the prelamin A substrate that are required for efficient cleavage by ZMPSTE24. We find that the C-terminal 41 amino acids of prelamin A contain sufficient context to allow cleavage of the tail by ZMPSTE24. We have identified several mutations in amino acids immediately surrounding the cleavage site (between Y646 and L647) that interfere with efficient cleavage of the prelamin A tail; these mutations include R644C, L648A and N650A, in addition to the previously reported L647R. Our data suggests that 9 of the 15 residues within the cleaved tail that lie immediately upstream of the CAAX motif are not critical for ZMPSTE24-mediated cleavage, as they can be replaced by the 9 amino acid HA epitope. However, duplication of the same 9 amino acids (to increase the distance between the prenyl group and the cleavage site) impairs the ability of ZMPSTE24 to cleave prelamin A.

**Conclusions/Significance:**

Our data reveals amino acid preferences flanking the ZMPSTE24 cleavage site of prelamin A and suggests that spacing from the farnesyl-cysteine to the cleavage site is important for optimal ZMPSTE24 cleavage. These studies begin to elucidate the substrate requirements of an enzyme activity critical to human health and longevity.

## Introduction

**Figure 1:**
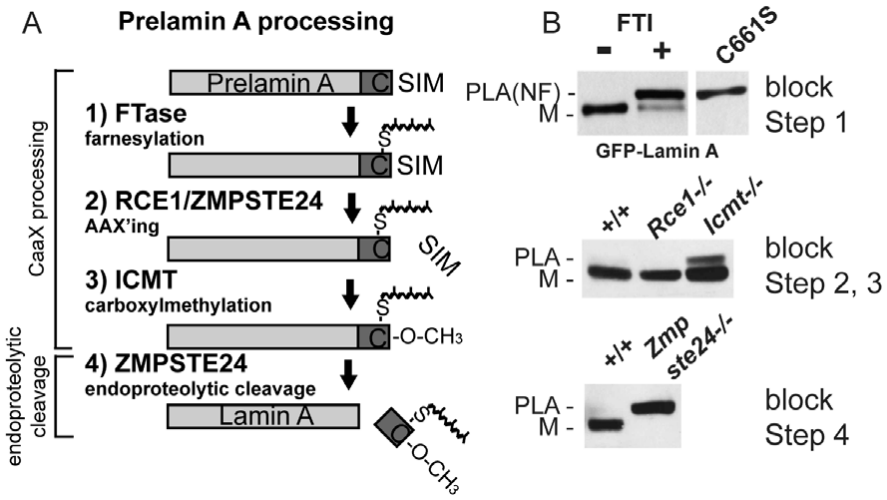
Assessing ZMPSTE24-mediated cleavage of the tail from prelamin A in cells blocked at different steps of CAAX processing. A) Schematic of the post-translational processing pathway for lamin A. Lamin A is synthesized as a precursor, prelamin A, and undergoes CAAX processing (farnesylation, AAX'ing, and carboxylmethylation; steps 1–3), followed by ZMPSTE24-mediated cleavage of the farnesylated, carboxylmethylated tail (step 4). B) An NIH 3T3 stable cell line expressing GFP-lamin A was treated with and without farnesyltransferase inhibitor (FTI). Lysates from these cells were compared to lysates prepared from NIH 3T3 stable cell line expressing GFP-lamin A with a mutation of the CAAX cysteine to serine (C661S) on an 8% SDS-PAGE gel (step 1 block). Lysates prepared from WT MEFs and MEFs containing knockouts of the indicated genes (*Rce1*, *Icmt*, and *Zmpste24*; S. Young, UCLA) were run on 8% gels and probed with antibodies to detect endogenous lamin A. The forms of lamin A are indicated: M (mature), PLA (prelamin A), and PLA(NF) (prelamin A, non-farnesylated).

The premature aging disease Hutchinson-Gilford Progeria Syndrome (HGPS) and related progeroid disorders are caused by defective processing of the lamin A precursor, prelamin A [1], [2]. Lamins are intermediate filament proteins that form a network of polymerized proteins that underlies the nuclear envelope. The nuclear lamins regulate the structure and shape of the nucleus, and provide an organizing platform for heterochromatin, transcription factors and nuclear pore complexes [3], [4]. There are two types of lamins, A-type (comprising the splice isoforms lamin A and C), and B-type (lamin B1, B2). All lamins, with the exception of lamin C, undergo post-translational modification of their C-terminal CAAX motif (in which C is cysteine, A is often aliphatic, and X is any residue), an event thought to be integral to their localization and function at the nuclear envelope [5], [6]. Modification of the CAAX motif involves an ordered series of events that includes farnesylation of the CAAX cysteine, endoproteolytic removal of the terminal three (AAX) residues, and finally carboxylmethylation of the farnesyl-cysteine. CAAX modification and the enzymes involved are shown for the lamin A precursor, prelamin A, in Figure 1A (steps 1–3). While the farnesyltransferase (FTase) is a soluble enzyme, the enzymes RCE1, ZMPSTE24 and ICMT are integral membrane proteins that reside in the endoplasmic reticulum (ER) and also in the inner nuclear membrane (INM) [6]–[8].

The hydrophobic post-translational modifications appended to the CAAX cysteine (farnesyl and carboxylmethyl groups) are thought to help anchor the lamins to the lipid bilayer of the INM, at least initially. However, while B-type lamins retain their farnesyl and methyl modifications, lamin A does not [9], [10]. Subsequent to CAAX modification of the C-terminus of prelamin A, the 15 amino acid long “tail” of prelamin A is endoproteolytically cleaved off, releasing mature lamin A (Figure 1A, step 4). The enzyme that mediates this cleavage step is ZMPSTE24, a multispanning membrane Zn^++^ metalloprotease [11], [12]. Prelamin A is the only known mammalian substrate for ZMPSTE24. Interestingly, ZMPSTE24 plays dual roles in lamin A maturation, both in 1) AAX'ing (endoproteolyis of the CAAX motif; Figure 1A, step 2), a role in which it is functionally redundant with RCE1, and in 2) tail cleavage, a role in which ZMPSTE24 is uniquely required (Figure 1A, step 4) [5], [13], [14].

The purpose of removing the modified tail from prelamin A is not clear, but failure to do so is associated with progeroid disorders ranging in severity from relatively mild to neonatal lethal. These diseases, in order of increasing severity, include metabolic disorder (MD) [15], mandibuloacral dysplasia type B (MAD-B) [16], Hutchinson-Gilford Progeria Syndrome (HGPS) [1], [2], [17], and the neonatal lethal restrictive dermopathy (RD) [18], [19]. These diseases are caused by mutations that map to genes encoding either lamin A, or the enzyme ZMPSTE24, that result in a partial or full block in cleavage of the CAAX-modified prelamin A tail. HGPS is the most well-known and best-studied of these diseases [17], [20], [21]. HGPS is a devastating premature aging disease resulting from a mutation that activates a cryptic splice site in one lamin A gene. The splicing error removes the coding region for the ZMPSTE24 cleavage site from prelamin A, resulting in the production of a mutant form of lamin A called progerin that is farnesylated and carboxylmethylated, but unable to be cleaved by ZMPSTE24. Children with HGPS begin to manifest symptoms of accelerated aging between 1–2 years of age, and often succumb to stroke at an average age of 13 years [22]. HGPS as well as the other progeroid disorders highlight the importance of the ZMPSTE24 cleavage of prelamin A for human health.

Inhibition of prelamin A processing by ZMPSTE24 may have additional relevance to human health. Certain protease inhibitor drugs that target the aspartyl protease of HIV have also been shown to inhibit ZMPSTE24, an undesirable side effect for which the mechanism remains unclear, and which may contribute to the lipodystrophy and metabolic disorder side effects experienced by patients who have used these medications as part of a long term treatment regimen [23]–[26]. In vascular smooth muscle cells from older individuals, ZMPSTE24 cleavage of prelamin A is diminished, which could contribute to the cardiovascular issues associated with normal physiological aging [27].

Because ZMPSTE24 activity is critical for human health and longevity, it is important to determine the substrate requirements of prelamin A cleavage by the ZMPSTE24 enzyme. Here, we have examined several aspects of the prelamin A substrate that are required for recognition and proteolysis by ZMPSTE24. We find that the last 41 amino acids of the C-terminus are sufficient for efficient cleavage, while 31 amino acids are not, and furthermore that particular residues within this region, including some in the vicinity of the cleavage site, contribute to recognition and cleavage by ZMPSTE24, while other residues do not appear to be critical. We also provide evidence that insertion of a spatial linker between the cleavage site and the farnesyl-carboxylmethyl cysteine can negatively impact cleavage efficiency. These studies begin to define the characteristics of an enzymatic cleavage event underlying several human progeroid disorders.

## Results

ZMPSTE24 plays an important role in cleaving the tail from prelamin A. However, little is known about substrate recognition except that farnesylation appears to be a critical requirement [9], [28]. Preventing ZMPSTE24 cleavage by inhibiton of CAAX processing can be achieved by the use of farnesyltransferase inhibitors (FTIs), or by mutation of the CAAX cysteine (C661S) (Figure 1B, top panel) [13], [29]–[32]. While the importance of farnesylation of the CAAX cysteine has been well-established, it has yet to be determined whether the farnesyl group is required specifically for ZMPSTE24 recognition, binding and/or cleavage, or in a more indirect manner for promotion of prelamin A membrane association where ZMPSTE24 resides. It is also important to note that farnesylation is required for all subsequent lamin A processing to occur, including endoproteolytic removal of the terminal AAX residues and carboxylmethylation.

To determine the impact on prelamin A cleavage due to lack of individual CAAX processing enzymes, we examined MEFs derived from mouse genetic knockouts of *Rce1* or *Icmt*, encoding the AAX'ing and carboxylmethylation enzymes, respectively, as compared to *Zmpste24* knockout MEFs. MEFs that are *Rce1−/−* are unaffected in prelamin A maturation (Figure 1B, middle panel), consistent with the observation that RCE1 and ZMPSTE24 are functionally redundant for this step (C. Hrycyna, S. Michaelis, unpublished). In MEFs that are *Icmt−/−*, the lack of carboxylmethylation modestly reduces the efficiency of prelamin A cleavage, but clearly does not significantly block it, in contrast to the case in *Zmpste24−/−* MEFs where cleavage is completely blocked (Figure 1; compare middle panel to bottom panel). This result suggests that while the farnesyl group is absolutely required for cleavage (Figure 1B top), the carboxylmethyl group is not essential, although the efficiency of cleavage by ZMPSTE24 is slightly reduced in its absence, as observed here and also noted elsewhere [6], [13], and may reflect a kinetic delay (Figure 1B, middle).

### The last 41 amino acids of prelamin A are sufficient for efficient cleavage by ZMPSTE24, while 31 amino acids are not

**Figure 2:**
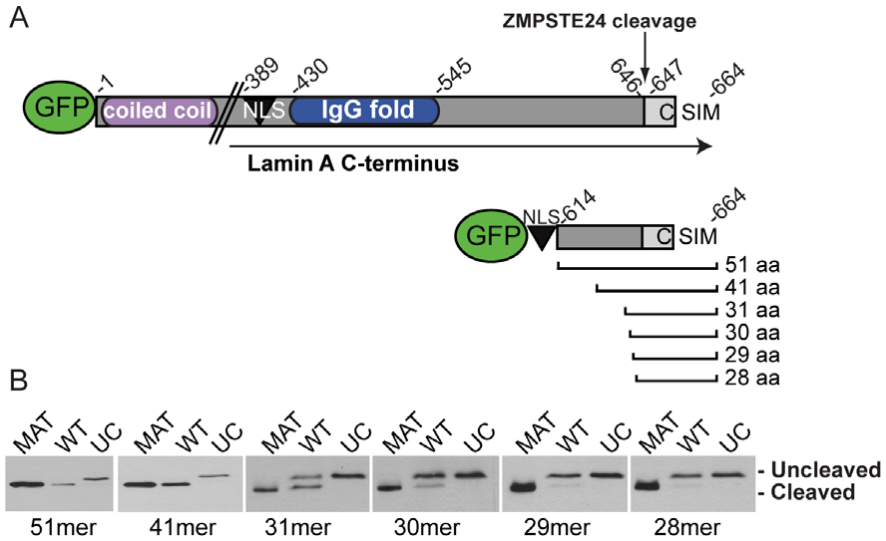
The last 41 amino acids of prelamin A are sufficient for efficient cleavage of prelamin A by ZMPSTE24. A) Schematic representation of full length prelamin A, and the deletion set analyzed here. Each deletion consists of GFP fused to the NLS of prelamin A (residues 416–423) and sequentially shorter C-terminal sequences (the cleaved tail encompasses the 15 residues 647–661; prior AAX'ing removes 662–664). B) Analysis of ZMPSTE24-mediated cleavage of the prelamin A deletion constructs. The deletion constructs were transiently transfected into HEK293A cells, and lysates were prepared 24 hours post-transfection. Proteins were resolved on 12% SDS-PAGE gels. For each deletion, mature (MAT) and uncleavable (UC; L647R) versions were included in the analysis to serve as markers for the migration of cleaved and uncleaved versions of each deletion, respectively.

We sought to identify the minimal region of the prelamin A C-terminus that contains all critical context for ZMPSTE24-mediated cleavage of the prelamin A tail. The full length prelamin A precursor is 664 amino acids long, and contains several defining features: an N-terminal globular head followed by coiled-coil domains, a nuclear localization sequence (NLS), an IgG fold, and a CAAX motif (Figure 2A). Previous work has shown that the coiled-coil domains (located within amino acids 1–388 of lamin A) are not required for CAAX processing and ZMPSTE24-mediated cleavage [33], [34].

We tested increasingly smaller segments of the C-terminus for cleavage by ZMPSTE24 by designing a series of C-terminal GFP-tagged lamin A constructs that contain GFP, the lamin A NLS, and increasingly smaller portions of the region between the NLS and the ZMPSTE24 cleavage site (Figure 2A). For each deletion construct, we also made mature (MAT) and uncleavable (UC) versions, with MAT containing a STOP codon after Y646 and UC containing a previously reported L647R mutation that blocks removal of the lamin A tail [35], to use as size controls for proper processing on our gels. We transfected the constructs into HEK293A cells and monitored cleavage to the mature form by Western blot analysis (Figure 2B). We found that the 51mer and 41mer constructs were efficiently converted to mature lamin A. Cleavage could still be detected with deletion of an additional 10 amino acids (31mer), however the efficiency of cleavage is reduced by about half in this context. Notably, deletion of two additional residues (29mer) effectively abolishes cleavage by ZMPSTE24 (Figure 2B). While it is possible that fusion to GFP may create a steric hindrance, and that even fewer residues may actually be required, we can conclude from our results that the complete context required for efficient ZMPSTE24 cleavage is contained within the last 41 amino acids of the prelamin A tail.

### Replacement of C-terminal residues does not block cleavage by ZMPSTE24

**Figure 3:**
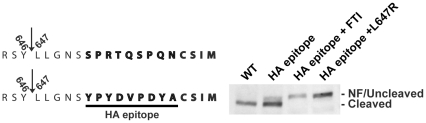
Replacing the nine C-terminal tail residues immediately preceding the CAAX motif does not significantly inhibit cleavage by ZMPSTE24. Within the GFP-51mer, nine amino acids immediately upstream of the CAAX motif (underlined) were replaced with a nine amino acid long HA epitope and transiently transfected into HEK293A cells. A construct including an uncleavable (UC; L647R) version of the HA swap construct was generated and included as a marker for migration for cleavage inhibition, in addition to FTI treatment. The NF (non-farnesylated) as well as the uncleaved and the cleaved forms are indicated.

It is unclear how the farnesyl group on the CAAX cysteine (and to a far lesser extent the carboxylmethyl group) can influence ZMPSTE24-mediated processing at its cleavage site 15 amino acids away. We wished to further probe whether other information within the C-terminal tail of prelamin A, or the length of the tail between the CAAX motif and the cleavage site, has an impact on ZMPSTE24 cleavage. We first investigated whether the prelamin A sequence immediately upstream of the CAAX motif is required for ZMPSTE24 cleavage. We utilized an epitope replacement strategy, convenient for introducing a random sequence while maintaining the distance between the CAAX motif and the cleavage site, by replacing nine amino acids immediately preceding the CAAX motif with the nine amino acid HA epitope (Figure 3). As a basis for the construct, we used the GFP-51mer, for which there is no defect in cleavage. We found that when the HA epitope was used to replace the C-terminal sequence, the cleavage of prelamin A was only very slightly impacted (Figure 3). Importantly, cleavage of the HA epitope replacement protein is blocked in the presence of FTI and in the L647R version of the HA replacement, indicating that the proteolytic cleavage we observe is ZMPSTE24-dependent. Since 9 of the 15 amino acids within the cleaved tail can be arbitrarily replaced without causing a significant impact on ZMPSTE24 cleavage, we conclude that these nine residues do not appear to be critical determinants for cleavage of prelamin A by ZMPSTE24.

### Increasing the distance between the CAAX box and the cleavage site reduces the cleavage efficiency of prelamin A

**Figure 4:**
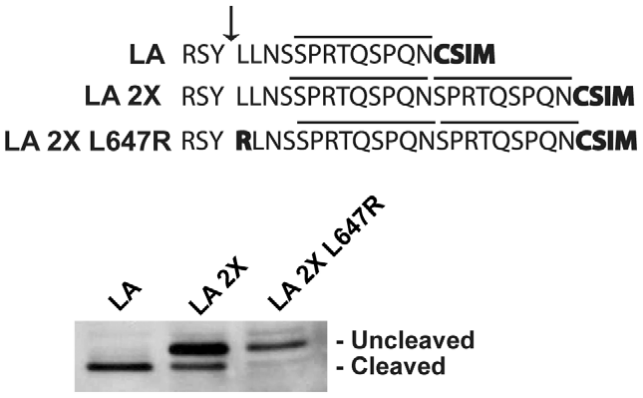
Increasing the spacing between the CAAX motif and the tail cleavage site affects prelamin A cleavage by ZMPSTE24. In order to increase the linear distance between the tail cleavage site and the CAAX cysteine, the nine amino acid sequence upstream of the CAAX motif (SPRTQSPQN) was duplicated within the GFP-51mer. A version of this construct bearing the uncleavable (UC; L647R) mutation was also generated as a marker for migration of uncleaved species. Constructs were transiently transfected into HEK293A cells and analyzed as in Figure 2.

We considered the possibility that ZMPSTE24 might have a “molecular reach” capacity, binding to a farnesylated and carboxylmethylated cysteine and cleaving within a certain distance away, as long as an allowable cleavage site is present. Thus, we investigated whether increasing the distance between the CAAX box and the cleavage site could impact cleavage by creating a construct that contained a duplication of the 9 “non-critical” amino acids we previously replaced with an HA epitope. This construct, designated LA 2X, showed a significant block in cleavage (albeit not 100% complete) (Figure 4). The residual cleavage that did occur must be ZMPSTE24-dependent, since the L647R mutation completely abolished it. Thus, increasing the length of the prelamin A tail interferes significantly with cleavage at the normal site. This result suggests a model for prelamin A cleavage in which ZMPSTE24 seeks an appropriate cleavage site within an allowable distance from the farnesyl-cysteine. It could also be the case that physical attributes of the duplicated region itself impede access to the motif (i.e. due to a physical barrier, created by secondary structure or rigidness). This possibility seems unlikely, however, since we have simply duplicated a region already present within the tail. In addition, we found that insertion of a myc epitope immediately upstream of the CAAX motif also significantly blocks cleavage (Figure S1). Regardless of the exact mechanism at work, we conclude that increasing the number of residues between the cleavage site and the CAAX motif can have a detrimental impact on cleavage efficiency by ZMPSTE24.

### Analysis of the residues surrounding the cleavage site by limited mutagenesis reveal a contextual requirement for proteolysis by ZMPSTE24

Most proteases recognize specific residues at, flanking, or near their cleavage site. ZMPSTE24 appears to recognize a specific cleavage site, based at least in part on sequence, as suggested by the L647R (UC) mutation. Importantly, our finding that cleavage is impaired by the presence of additional residues between the farnesyl-cysteine and the cleavage site suggests that ZMPSTE24 does not simply cleave a certain distance from the CAAX motif, and thus is not simply a “molecular ruler”.

**Figure 5:**
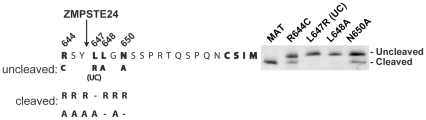
Mutagenesis of the residues surrounding the tail cleavage site reveals a contextual requirement and evidence for a consensus motif for the cleavage of prelamin A by ZMPSTE24. The indicated residues upstream and downstream of the cleavage site (between Y646 and L647) within the GFP-51mer were mutated to either alanine (A) or arginine (R) and assayed for cleavage by ZMPSTE24 by transient transfection into HEK293A cells. The R644C mutation was also included based on its reported presence in atypical progeria and other diseases as discussed in the text. Amino acid changes that do and do not impact cleavage are indicated. Gel analysis of mutants that impair cleavage is shown.

In order to find additional residues that may contribute to cleavage specificity, we performed a limited mutagenesis analysis on the region immediately flanking the Y646 and L647 cleavage site residues. We mutated residues R644 to S651 in the GFP-51mer to either arginine (R) or alanine (A), and assayed for the ability of ZMPSTE24 to cleave the lamin A tail by transient transfection of HEK293A cells with these constructs, run side by side with mature (MAT) and L647R (UC) versions as controls (Figure 5). In addition to the previously established L647R (UC), we found L648A and N650A as additional mutations that affect cleavage of the tail by ZMPSTE24. L648A completely blocks tail cleavage, while N650A is only partially defective for cleavage. None of the other R or A mutations tested block cleavage of the tail (Figure 5, and data not shown). It is notable that it is not simply the residue, but the particular amino acid change that is made that appears to be important, (i.e. L648R is unaffected, while L648A is blocked in cleavage). Thus, a more comprehensive mutagenic analysis will be required to fully elucidate the sequence motif that is recognized by ZMPSTE24.

The R644 residue is mutated to cysteine in several human diseases with diverse phenotypes, including a case of atypical progeria [36]–[38]. Thus, the R644 residue may be important for promoting cleavage by ZMPSTE24. Because the R644A mutation did not affect cleavage in our assay, we recreated the R644C disease mutation and examined the ability of this mutant to be cleaved. Interestingly, we find a partial cleavage defect when the GFP-51mer containing the R644C mutation is expressed in HEK293A cells (Figure 5). The partial inhibition of cleavage that results from this mutation suggests that R644 is an important contributing residue for efficient ZMPSTE24 cleavage, along with L648 and N650.

## Discussion

The proteolytic processing and cleavage of the prelamin A tail is critical for lamin A function. Several progeroid disorders result from mutations in the genes encoding lamin A or ZMPSTE24 that interfere with the final proteolytic event that removes the modified tail [14], [17], [39]–[41]. The symptoms associated with these diseases range in severity and appear to correlate with the extent of the lamin A cleavage defect such that the greater the amount of uncleaved persistently prenylated prelamin A that accumulates, the more severe the disease. For example, a partial reduction in ZMPSTE24 activity results in diseases that manifest later than HGPS (MAD-B) [42], [43], while mutations that more extensively impact prelamin A cleavage result in the accelerated aging phenotype of the childhood progeria disease HGPS [44]. The complete loss of any ZMPSTE24 cleavage activity results in neonatal lethality (RD), thus underscoring the vital importance of ZMPSTE24 to human health [19], [45].

The permanently prenylated form of lamin A appears to confer a dominant negative function to lamin A, altering its membrane binding characteristics and negatively impacting many nuclear functions. Thus, drug therapeutic intervention strategies for HGPS have been designed to target and inhibit the prenylation of prelamin A by the use of FTIs [46]. The goal of the drug therapy is to prevent the addition of the hydrophobic CAAX modifications and thus reduce the toxicity of the aberrant protein. It is clear that an understanding of the ZMPSTE24-mediated proteolytic event that normally removes the prenylated lamin A tail is critical to inform future therapeutic strategies, in particular for those diseases resulting from mutations in ZMPSTE24 itself that impair enzymatic activity. Lack of ZMPSTE24-mediated cleavage of prelamin A may also contribute to normal physiological aging, thus further highlighting the importance of understanding the molecular details of this processing step [27].

**Figure 6:**
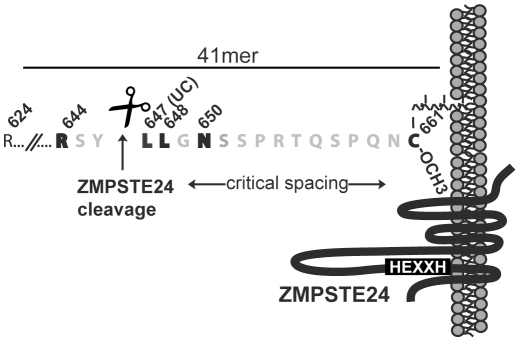
Summary of the critical features found in this study for ZMPSTE24-mediated cleavage of the prelamin A tail. Within the terminal 41 amino acids (residues 624–664) shown here to be needed for efficient cleavage (Figure 2), three new residues in addition to L647 (L647R = UC) were identified in this study that contribute to an apparent sequence requirement in the region of the cleavage site (R644, L648, and N650) (Figure 5). The identity of the nine amino acids (SPRTQSPQN) upstream of the CAAX motif appears not to be critical for cleavage (Figure 3). Placing the cleavage site at a further distance from the CAAX motif was found to inhibit cleavage by ZMPSTE24 (Figure 4), suggesting that a critical length from the farnesyl-cysteine may be tolerated by ZMPSTE24. Farnesylation is critical for further processing and cleavage by ZMPSTE24, while carboxylmethylation only modestly contributes to cleavage efficiency (Figure 1).

Despite the importance of enzymatic cleavage of the prelamin A tail, ZMPSTE24 activity toward prelamin A remains largely uncharacterized. Sequence alignments with multiple lamin A homologues known to undergo cleavage (human, mouse, and chicken) [12], [33], [35] reveal that the region surrounding the cleavage site is well-conserved (Figure S2). In this study we have examined several essential features of ZMPSTE24-mediated prelamin A tail cleavage, and our results are summarized in Figure 6. We found that the 41 amino acid tail of prelamin A is sufficient for efficient ZMPSTE24 cleavage (Figure 2), and that mutation of certain residues near the cleavage site can influence cleavage (Figure 5). Importantly, for each of the residues for which a particular change partially or completely blocked cleavage by ZMPSTE24 (R644, L647, L648 and N650), we found another change(s) to have no effect. The fact that a certain residue at a particular position relative to the cleavage site is allowable while another is not (i.e. L648R is cleaved, while L648A is not) suggests that cleavage is not strictly dependent on the presence of a particular amino acid and that a consensus motif may remain to be defined. We have also found that increasing the distance between the CAAX motif and the cleavage site reduces cleavage efficiency (Figure 4). This result argues against the possibility that ZMPSTE24 may simply act as a “molecular ruler” that cleaves a measured distance from the CAAX motif.

Two models can be envisioned to explain such an additional “spatial requirement” between two important substrate features (the prenylated cysteine and the cleavage site). First, the CAAX modifications promote membrane association of the prelamin A substrate. It is possible that ZMPSTE24, which is a transmembrane protein with its HEXXH zinc metalloprotease domain in a cytosolic/nucleoplasmic loop [14], can interact with the prelamin A cleavage site only within a limited distance from the site of membrane attachment of prelamin A through its farnesylated cysteine (Figure 6). Thus, the addition of sequence between the CAAX motif and the cleavage site may result in the cleavage site being located outside of the “membrane-tethered reach” of ZMPSTE24. Alternatively, ZMPSTE24 may directly recognize and bind to the farnesyl-carboxylmethyl cysteine at one internal binding site within ZMPSTE24, and cleave prelamin A using an active site elsewhere in ZMPSTE24. Thus the spatial restriction found in this study may reflect the internal “molecular reach” between the farnesyl recognition/binding site within ZMPSTE24 and the active proteolytic site within ZMPSTE24 that carries out cleavage. It will be of interest to determine whether decreasing the distance between the cleavage site and the CAAX motif would impact ZMPSTE24 cleavage. There is currently no evidence for separate farnesyl-binding and active sites for ZMPSTE24, and whether farnesyl is required for cleavage directly (for binding between prelamin A and the enzyme), or indirectly (for proper membrane positioning of the prelamin A substrate with the enzyme active site) remains to be determined.

The sole residue residue examined in our mutagenesis analysis for which mutations are associated with human diseases is R644. The R644C mutation is associated with pleiotropic disease phenotypes, and has been identified in cases of atypical progeria and lipodystrophy, as well as motor neuropathy, limb girdle muscle weakness and dilated cardiomyopathy [37]. The R644H mutation has also been reported in a case of muscular dystrophy [47]. The association of R644C with atypical progeria and lipodystrophy is particularly intriguing, since both disease phenotypes can result from a failure to cleave lamin A. Given the potential molecular mechanism that connects the range of progeroid disorders, namely failure to cleave prelamin A, we investigated whether prelamin A could be detected in fibroblasts from a patient with atypical progeria with the R644C mutation (Coriell Institute; AG00989). We found that while a very small amount of prelamin A was detectable, the amount in these cells was variable between experiments, and we did not consistently observe a significant difference from wild type (data not shown). It is worth noting that the disease phenotypes associated with R644C are variable and mild compared to progeria, and it remains possible that prelamin A processing could be affected in patients in a tissue-specific manner, and thus not able to be detected in the fibroblast sample available. Prelamin A also accumulates over time in wild type cell lines with increased passaging, thus it remains an open question whether the amount present in the patient cells would significantly correlate with disease pathology.

## Materials and Methods

### Cell Culture Conditions

NIH 3T3 fibroblasts, HEK293A, and mouse embryonic fibroblasts (MEFs) (*Zmpste24−/−*, *Rce1−/−*, *and Icmt−/−* obtained from S. Young, UCLA) [12], [48], [49], were maintained in DMEM (GIBCO) supplemented with 10% fetal bovine serum (GIBCO). Farnesyltransferase inhibitor R115777 (a gift from M. Gelb, University of Washington) was used in cell culture medium at a final concentration of 1 µM for 24 h.

### Stable and Transient Expression in Cell Culture

NIH 3T3 fibroblasts stably expressing GFP-lamin A and GFP-lamin A C661S TetOff constructs were created by retroviral transduction, as described previously [6]. Retrovirus was prepared by transient co-transfection of HEK293F cells with the packaging plasmid pCL-Eco using Fugene 6 Transfection Reagent (Roche Applied Science, Indianapolis, IN) according to the manufacturer's protocol. All other constructs in this study were expressed by transient transfection of a HEK293A TetOff cell line, using Fugene 6. Lysates for gel analysis were prepared 24 hours post-transfection.

### Plasmid Constructs

All plasmids were created in the pSM2277 backbone, a retroviral pMX vector containing an inducible Tet promoter and a hygromycin selection cassette. GFP-lamin A (pSM 2278) and GFP-lamin A bearing the C661S mutation (pSM2308) were described previously [6]. The series of lamin A deletion constructs shown in Figure 2 were constructed by fusing the NLS from lamin A (amino acids 416–423) to the C-terminus of GFP, to which was appended increasingly shorter lengths of the lamin A C-terminus. For each deletion, an uncleavable (UC) version (L647R) and a mature version (STOP codon after Y646) were also generated. The HA epitope, myc epitope, duplicated lamin A region (LA 2X), and various lamin A mutations were all introduced into the GFP-51mer (pSM2478) using standard cloning techniques and the QuikChange Site Directed Mutagenesis Kit (Stratagene, La Jolla, CA) according to the manufacturer's instructions. The nine amino acid HA epitope was inserted in frame to replace lamin A residues 652–660, while the LA 2X construct was created by inserting the sequence from 652–660 between residues 660 and 661, to create a duplicated nine amino acid region immediately upstream of the CAAX motif. Single point mutations were introduced using the QuikChange Site Directed Mutagenesis Kit.

### Western Blotting and Antibodies

Cell lysates were made by direct lysis in SDS-PAGE sample buffer followed by heating to 65°C for 10 min, and then were briefly sonicated to disrupt nuclear chromatin. For detection of full length GFP-lamin A, GFP-lamin A C661S, and endogenous lamin A in MEFs (Figure 1), lysates were resolved on 8% SDS-PAGE gels. For detection of the lamin A deletions, the HA epitope replacement, the lamin A duplication (LA 2X), and the lamin A mutants, the cell lysates were resolved on 12% SDS-PAGE gels (Figures 2, 3, 4, and 5). Gels were transferred to nitrocellulose and blocked using TBST (100 mM Tris-HCl, pH 8.0, 150 mM NaCl, 0.05% Tween-20) and 10% Western Blocking Reagent (Roche Applied Science, Indianapolis, IN). Polyclonal antibodies to lamin A (Santa Cruz, sc-6214) or a monoclonal antibody to GFP (Roche) were used at 1∶1000. Secondary antibodies used included HRP conjugated anti-rabbit and anti-mouse (GE Healthcare, UK). Secondary antibodies were detected using Amersham ECL Plus Western Blotting Detection System (GE Healthcare, UK) according to the manufacturer's instructions. All Western blot experiments were performed at least in triplicate, and a representative example is shown.

## Supporting Information

Figure S1 **Insertion of a myc epitope between the CAAX motif and the tail cleavage site prevents prelamin A cleavage by ZMPSTE24.** The 10 amino acid long myc epitope was inserted immediately upstream of the CAAX motif, to create “LA Mycins”. Constructs were transiently transfected into HEK293A cells and analyzed by SDS-PAGE as in Figure 2. Uncleavable (UC) and wild type (LA) versions were included as markers for the migration of uncleaved and mature species, respectively. As an additional control for the migration of the uncleaved LA Mycins species, 1 uM FTI was included to prevent the farnesylation and cleavage of LA Mycins (last lane).
(TIF)

Figure S2 **Sequence alignment of human, mouse and chicken lamin A homologues for the 41mer region examined in this study.** Sequences have been reported for mammals, birds and frogs, all of which show a substantial degree of conservation, with some drift in frogs. The sequences shown here correspond to those for which cleavage of the lamin A tail has been experimentally demonstrated [12], [33], [35]. The sequence comparison (made using ClustalW and Boxshade) is shown with identical residues shaded in black, and conserved residues in gray. The lamin A residues found in this study to be critical for prelamin A cleavage are outlined in red. Likewise, the region that can be replaced with the HA epitope without affecting cleavage is also noted.
(TIFF)
