# Supplementary figures and images for: Requirements for Efficient Proteolytic Cleavage of Prelamin A by ZMPSTE24
Source: PLoS One. 2012 Feb 15;7(2):e32120. doi: 10.1371/journal.pone.0032120 (PMC3280227; doi:10.1371/journal.pone.0032120)

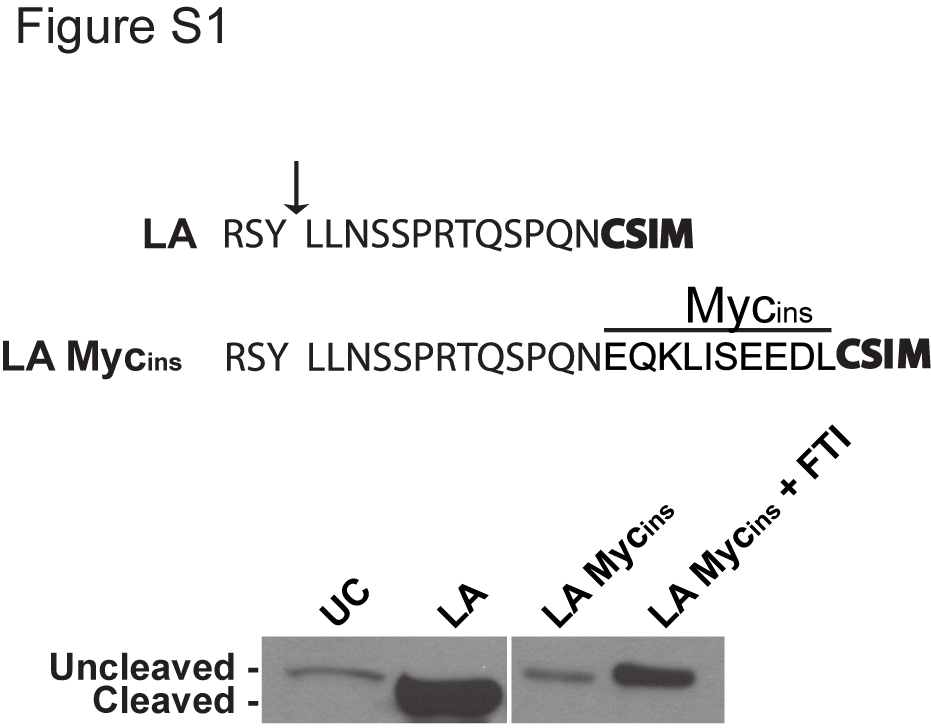

Supplement: Figure S1 — Insertion of a myc epitope between the CAAX motif and the tail cleavage site prevents prelamin A cleavage by ZMPSTE24. The 10 amino acid long myc epitope was inserted immediately upstream of the CAAX motif, to create “LA Mycins”. Constructs were transiently transfected into HEK293A cells and analyzed by SDS-PAGE as in Figure 2. Uncleavable (UC) and wild type (LA) versions were included as markers for the migration of uncleaved and mature species, respectively. As an additional control for the migration of the uncleaved LA Mycins species, 1 uM FTI was included to prevent the farnesylation and cleavage of LA Mycins (last lane). (TIF) [file pone.0032120.s001.tif]

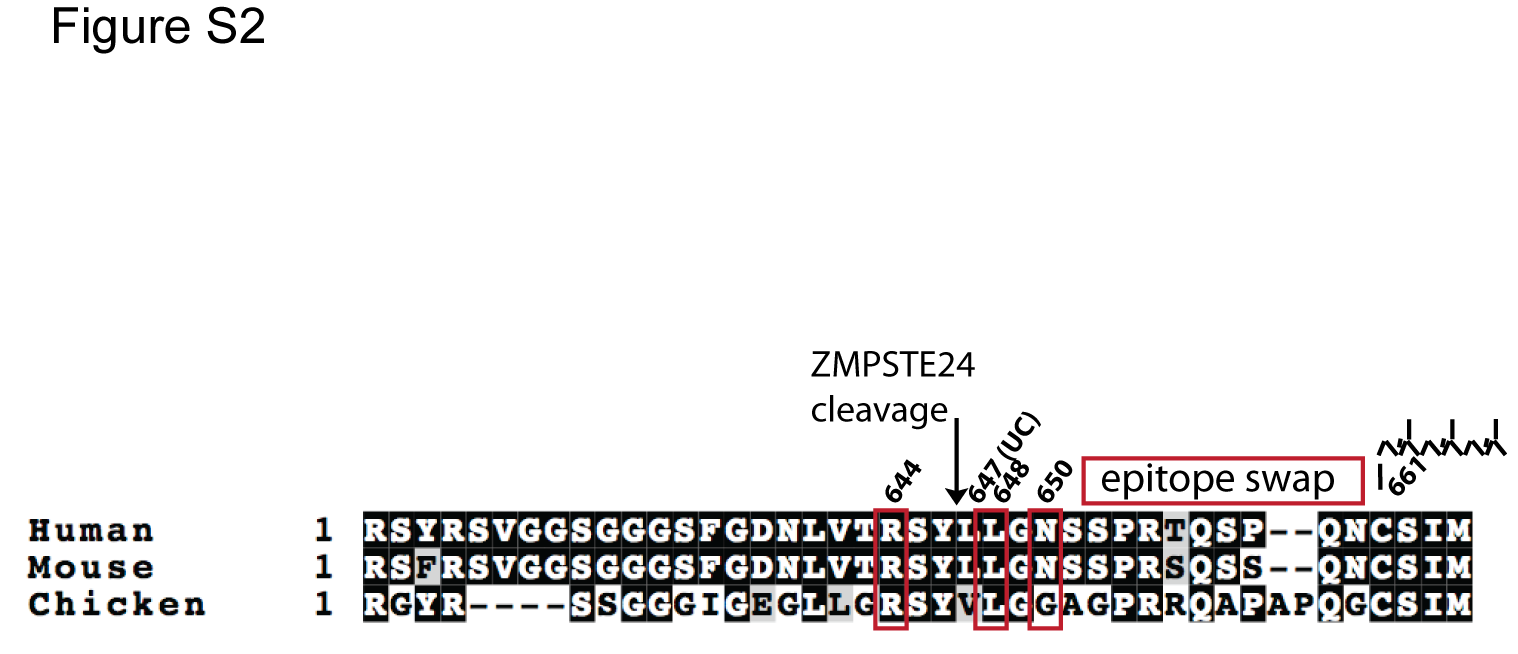

Supplement: Figure S2 — Sequence alignment of human, mouse and chicken lamin A homologues for the 41mer region examined in this study. Sequences have been reported for mammals, birds and frogs, all of which show a substantial degree of conservation, with some drift in frogs. The sequences shown here correspond to those for which cleavage of the lamin A tail has been experimentally demonstrated [12], [33], [35]. The sequence comparison (made using ClustalW and Boxshade) is shown with identical residues shaded in black, and conserved residues in gray. The lamin A residues found in this study to be critical for prelamin A cleavage are outlined in red. Likewise, the region that can be replaced with the HA epitope without affecting cleavage is also noted. (TIFF) [file pone.0032120.s002.tif]
